# Empfehlungen für ECMO bei COVID-19-Patienten: Consensus Paper zur Extrakorporalen Membranoxygenierung der Medizinischen Universität Wien

**DOI:** 10.1007/s00740-020-00349-x

**Published:** 2020-06-10

**Authors:** Dominik Wiedemann, Martin H. Bernardi, Klaus Distelmaier, Georg Goliasch, Christian Hengstenberg, Alexander Hermann, Michael Holzer, Konrad Hoetzenecker, Walter Klepetko, György Lang, Andrea Lassnigg, Günther Laufer, Ingrid A. M. Magnet, Klaus Markstaller, Martin Röggla, Bernhard Rössler, Peter Schellongowski, Paul Simon, Edda Tschernko, Roman Ullrich, Daniel Zimpfer, Thomas Staudinger, COVID-19 ECMO Arbeitsgruppe Medizinische Universität Wien

**Affiliations:** 1 Klinische Abteilung für Herzchirurgie, Universitätsklinik für Chirurgie, Allgemeines Krankenhaus der Stadt Wien, Medizinische Universität Wien, Währinger Gürtel 18–20, 1090 Wien, Österreich; 2 Allgemeine Intensivmedizin und Schmerztherapie, Klinische Abteilung für Herz-Thorax-Gefäßchirurgische Anästhesie und Intensivmedizin, Universitätsklinik für Anästhesie, Medizinische Universität Wien, Wien, Österreich; 3 Universitätsklinik für Innere Medizin II, Klinische Abteilung für Kardiologie, Medizinische Universität Wien, Wien, Österreich; 4 Universitätsklinik für Innere Medizin I, Allgemeines Krankenhaus der Stadt Wien, Medizinische Universität Wien, Währinger Gürtel 18–20, 1090 Wien, Österreich; 5 Universitätsklinik für Notfallmedizin, Medizinische Universität Wien, Wien, Österreich; 6 Klinische Abteilung für Thoraxchirurgie, Universitätsklinik für Chirurgie, Medizinische Universität Wien, Wien, Österreich; 7 Allgemeine Intensivmedizin und Schmerztherapie, Universitätsklinik für Anästhesie, Medizinische Universität Wien, Wien, Österreich; 8 Medizinisches Simulationszentrum, Universitätsklinik für Anästhesie, Wien, Österreich

**Keywords:** Akutes Atemnotsyndrom, Empfehlungen, Kriterien, Indikationen, Kanülierung, Acute Respiratory Distress Syndrome, Recommendations, Criteria, Indications, Canulation

## Abstract

Die aktuelle COVID-19-Pandemie, die durch das SARS-CoV‑2 ausgelöst wird, hat bereits in vielen betroffenen Ländern zu gravierenden Ressourcenengpässen der jeweiligen Gesundheitssysteme geführt. Obwohl sehr viele COVID-19-Patienten nur moderate Symptome zeigen, entwickelt eine Subgruppe ein schweres respiratorisches oder auch kardiales Versagen. Die extrakorporale Membranoxygenierung (ECMO) stellt eine wertvolle Therapieoption für Patienten mit therapierefraktärem Lungen- oder Herzversagen dar. Es bedarf klarer Empfehlungen, die den ECMO-Einsatz in dieser zahlenmäßig stetig steigenden Patientenpopulation regeln. Die ECMO-Arbeitsgruppe der Medizinischen Universität Wien hat daher die folgenden Empfehlungen für eine ECMO-Unterstützung bei COVID-19-Patienten formuliert.

## Abstract (en)

The pandemic from the SARS-CoV‑2 Virus is currently challenging health care systems all over the world. Maintaining appropriate staffing and resources in healthcare facilities is essential to guarantee a safe work environment for healthcare personnel and safe patient care. Extracorporeal membrane oxygenation (ECMO) represents a valuable therapeutic option in patients with severe heart or lung failure. Although only a limited proportion of COVID-19 patients develops respiratory or circulatory failure that is refractory to conventional therapies, it is of utmost importance to clearly define criteria for the use of ECMOs in this steadily growing patient population. The ECMO working group of the Medical University of Vienna has established the following recommendations for ECMO support in COVID-19 patients.

## Hintergrund

Die aktuelle COVID-19-Pandemie, die durch das SARS-CoV‑2 ausgelöst wird, hat bereits in vielen betroffenen Ländern zu gravierenden Ressourcenengpässen in den jeweiligen Gesundheitssystemen geführt [1].

Obwohl sehr viele COVID-19-Patienten nur moderate Symptome zeigen, entwickelt eine Subgruppe ein schweres respiratorisches Versagen unter dem Bild eines Acute Respiratory Distress Syndrome (ARDS) [2, 3]. Ebenso wird eine kardiale Beteiligung mit fulminanter Myokarditis, Herzrhythmusstörungen und Kreislaufversagen beschrieben [3, 4]. COVID-19-Patienten, die mechanisch beatmet werden müssen, weisen eine hohe Mortalität auf. Trotz der Tatsache, dass sowohl in China als auch in Italien (den Ländern mit den zum Zeitpunkt der Manuskripterstellung höchsten Zahlen an COVID-19-Patienten) die Anzahl an ECMO(Extrakorporale Membranoxygenierung)-Patienten unter den COVID-19-Fällen gering ist, erscheint es wichtig, sich in Österreich bzw. in Wien mit diesem speziellen Szenario auseinanderzusetzen und Richtlinien für den ECMO-Einsatz in diesem speziellen Fall zu erstellen.

Grundsätzlich darf bei weiterführenden Fragen auf die rezente Stellungnahme der European Life Support Organization (ELSO) verwiesen werden [5].

## Allokation

### Grundsatzfrage: Sollte eine ECMO-Therapie für COVID-19-Patienten in Betracht gezogen werden?

Derzeit befinden wir uns in Österreich noch in keiner Triagesituation, in der gewisse Patientengruppen grundsätzlich von Therapien ausgeschlossen werden. Dennoch muss eine Indikationsstellung zum ECMO-Support bei COVID-19 mit Rücksichtnahme auf mögliche zukünftige Ressourcenengpässe restriktiv gestellt werden [6]. Dies bedeutet aber nicht, dass Covid-19-Patienten generell von einer ECMO-Therapie ausgeschlossen werden sollen. Allerdings sind fortgeschrittenes Patientenalter, Komorbiditäten und grundsätzliche Prognoseeinschätzung wichtige Parameter, die bereits jetzt in die Entscheidung, COVID-19-Patienten mittels ECMO zu unterstützen, einfließen müssen.

Sollte es in Österreich zu einer Triagesituation ähnlich zu der derzeitigen Lage in Italien, Spanien und anderen Ländern kommen, ist die Entscheidung zur ECMO an die jeweilige Triageentscheidung der Intensivstation bzw. Ressourcenkapazitäten zu knüpfen.

An diese Grundsatzdebatte ist die Frage anzuschließen, ob in der Pandemiezeit neue ECMO-Zentren mit Implantationen beginnen sollten. Davon ist aus Zentrumssicht abzuraten, da einerseits die Ergebnisse in Zentren mit hohen Fallzahlen entsprechend zu favorisieren sind, andererseits das Ansteckungsrisiko für medizinisches Personal insbesondere am Beginn der Lernkurve höher erscheint. Gerade in Pandemiezeiten muss der Schutz des medizinischen Personals oberste Maxime sein.

### ECMO für Nicht-COVID-19-Patienten während der Pandemie

Auch hier ist anzumerken, dass derzeit in Österreich keine Triagesituation besteht, sodass die Entscheidung, einen Patienten an die ECMO zu nehmen, analog zu den bisherigen Therapieentscheidungen zu fällen ist. Sollte die COVID-19-Pandemie allerdings zu gröberen Ressourceneinschränkungen führen, kann es notwendig sein, auch hier restriktivere Entscheidungen treffen zu müssen, da die resultierende Ressourcenknappheit diese erfordern wird.

### Priorisierung von Patienten

Gerade in Pandemiezeiten erscheint ein Priorisieren der Patienten unabdingbar. „First come – first serve“-Entscheidungen müssen vermieden werden, da dies ansonsten darin endet, dass Patienten mit schlechter Prognose die Ressourcen für Patienten mit potenziell guter Prognose verbrauchen. Dieser Punkt ist auch zum momentanen Zeitpunkt (ohne Triagesituation) bereits beachtenswert, da schon jetzt im Hinblick auf zukünftige Engpässe Entscheidungen gefällt werden müssen. Die höchste Priorität soll für jüngere Patienten ohne Komorbiditäten gelten.

Da sich die Kapazitäten im Laufe der Pandemie dynamisch verändern, sind auch Priorisierungsentscheidungen dynamisch zu fällen und zu verändern. Diese sind im Extremfall sogar von Schicht zu Schicht anzupassen.

## Vorgehen und Ausschlusskriterien bei COVID-19-positiven ECMO-Kandidaten

Wenn sich ein SARS-CoV-2-positiver Patient außerhalb des Allgemeinen Krankenhauses (AKH) Wien anhand der gegebenen Richtlinien für eine ECMO qualifiziert, ist eine frühzeitige Kontaktaufnahme mit den intensivmedizinischen Koordinatoren des AKH Wien aus den Bereichen der Anästhesie oder Innere Medizin nötig.

### Protektive Maßnahmen

Für die entsprechenden Schutzmaßnahmen bei der ECMO-Implantation bzw. generell während der Behandlung von Patienten mit COVID-19 gelten die Richtlinien des Wiener Krankenanstaltenverbunds [7]. Zusätzliche Empfehlung für den Operateur zur Vermeidung einer Wundkontamination ist eine FFP-2- oder FFP-3-Maske ohne Ventil. Alternativ kann auch eine FFP-2- oder FFP-3-Maske mit Ventil mit einer konventionellen Mund-Nasen-Maske kombiniert werden.

### Ausschlusskriterien für eine ECMO-Therapie bei COVID-19-Patienten

Auf Basis der vorhandenen Ressourcen können sich die klinischen Ausschlusskriterien dynamisch ändern. Tab. 1 fasst absolute und relative Kontraindikationen für einen ECMO-Support bei COVID-19-Patienten zusammen. Aus derzeitiger Sicht sollten Patienten mit diesen Kriterien für einen ECMO-Support ausgeschlossen oder zumindest sehr kritisch evaluiert werden.

**Table 1:** 

Absolute Kontraindikationen	Relative Kontraindikationen
Ablehnung durch Patient	Alter >65 a^a^ (abhängig vom biologischen Alter)
Schweres vorbestehendes neurologisches Defizit, fortgeschrittene Demenz	Beatmungsdauer vor ECMO >7 Tage
Terminale Systemerkrankung (Lebenserwartung <1 Jahr)	Aktive relevante Immunsuppression
Aktive Hirnblutung	Hämatologische Systemerkrankung
Alter >75 Jahre oder Alter >70 plus ≥2 relative Kontraindikationen^a^	Zusätzliches Organversagen (außer Niere)
Weit fortgeschrittene terminale Lungenerkrankungen	Frailty [19]
Metastasiertes Malignom	Hochgradige Aorteninsuffizienz (für VA ECMO)
Leberzirrhose Child C	Schwere PAVK (für VA ECMO)
<1 Jahr nach allogener Stammzelltransplantation (PBSCT)	Chronische Herzinsuffizienz NYHA IV (ohne HTX- oder VAD-Option)

^a^Alterslimits müssen im Laufe der Pandemie ggf. adaptiert werden

## Venovenöse ECMO („respiratory ECMO“)

### Grundsatz.

ECMO bei COVID-19 ist eine *Rescuetherapie*, wenn die mechanische Beatmung den Gasaustausch nicht mehr gewährleisten kann, sodass eine lebens- oder organbedrohliche Hypoxie und/oder Hyperkapnie resultiert.

Eine ECMO-Therapie soll auch dann als indiziert angesehen werden, wenn die Invasivität und damit die negativen Effekte einer mechanischen Beatmung als zu hoch erachtet werden. Hier ist besonders auf den rechtzeitigen Einsatz bzw. die rechtzeitige Vorstellung des Patienten hinzuweisen.

### Indikationen und Kontraindikationen

**Figure 1:**
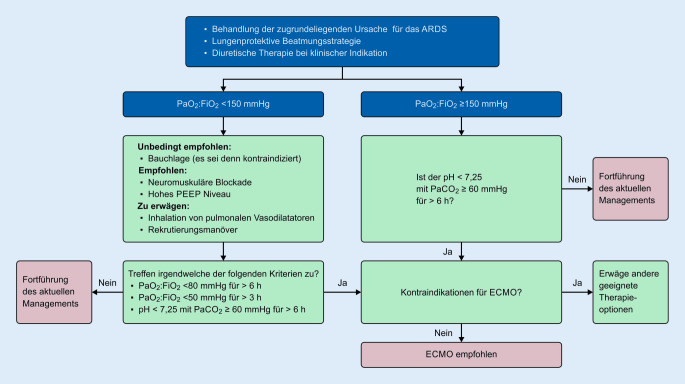

Als Grundlage für die Beurteilung, ob eine solche Situation vorliegt, kann der von der Extracorporeal Life Support Organization empfohlene Algorithmus unter den Bedingungen einer *optimierten Beatmungstherapie* herangezogen werden (Abb. 1; [5, 8]).

Um sekundäre Organschäden rechtzeitig zu verhindern, empfehlen wir eine bereits frühzeitige Erwägung einer ECMO-Therapie. In Abhängigkeit vom zeitlichen Verlauf des klinischen Zustandes sollte eine Vorstellung zur ECMO bereits bei zunehmender respiratorischer Verschlechterung (PaO_2_/FiO_2_ Ratio von <150) in Betracht gezogen werden.

#### Bemerkungen

- Die absoluten Kontraindikationen beziehen sich dezidiert auf Patienten mit schwerem Lungenversagen aufgrund einer SARS-CoV-2-Infektion.
- Die relativen Kontraindikationen beschreiben Faktoren, die bei Patienten mit Bedarf an respiratorischer ECMO mit einem schlechteren Outcome assoziiert sind. Bei Vorliegen relativer Kontraindikationen ist eine ECMO-Therapie nur in begründeten Ausnahmefällen zu erwägen!
- Für jeden Punkt bedarf es einer individuellen Abwägung.
- Das Vorhandensein mehrerer relativer Kontraindikationen verschlechtert die Prognose substanziell.
- Relative Kontraindikationen sind immer auch in Abhängigkeit von der Verfügbarkeit von Ressourcen zu betrachten.
- „Ideale“ ECMO-Kandidaten wären Patienten <65 Jahre ohne Vorerkrankungen und mit einem Ein-Organ-Versagen (Lunge).

### Kanülierung

- Wir empfehlen die Kanülierungsstrategie, mit der das Zentrum die besten Erfahrungen hat, z. B. eine femorojuguläre Konfiguration.
- Die Kanülengröße ist entsprechend des zu erwartenden Blutflussbedarfes zu wählen (Ziel: ECMO-Blutfluss >60 % des Patienten-HZV).
- Nachdem bei COVID-19-Patienten in erster Linie die schwersten Lungenversagen für ECMO qualifizieren („Rescue-ECMO“) ist mit der Notwendigkeit eines fast vollständigen Lungenersatzes zu rechnen, weshalb wir eine möglichst große Drainagekanüle empfehlen (bei normal großen Erwachsenen mit einem im Ultraschall gemessenen Venendurchmesser von ≥10 mm mindestens 23 F, besser 25 F).
- Die Reperfusionkanüle wird in der Regel im Durchmesser 2–4 F kleiner gewählt.

### Beatmungsstrategie unter VV ECMO

Unter VV ECMO ist eine lungenprotektive Beatmungsstrategie unter Berücksichtigung einer Reduktion von Tidalvolumen, „driving pressure“ und Atemfrequenz zu empfehlen [9–11]. Die PEEP-Titration sollte individuell entsprechend des Rekrutierungspotenzials erfolgen [12].

## Venoarterielle ECMO bei COVID-19-Patienten („cardiac ECMO“)

Obwohl bei COVID-19 grundsätzlich die pulmonale Beteiligung im Vordergrund steht, reicht bei einigen Patienten eine VV ECMO nicht aus. Der Grund dafür ist zum einen, dass manche Patienten zusätzlich zu einem pulmonalen Versagen ein konsekutives Kreislaufversagen entwickeln, zum anderen kommt es in einer Subgruppe der COVID-19-Patienten aber auch zu einer kardialen Beteiligung mit Arrhythmien oder Myokarditis. Es ist daher unbedingt notwendig, neben den üblichen hämodynamischen Überwachungsmaßnahmen bei COVID-19-Patienten im Rahmen einer ECMO-Evaluierung eine Echokardiographie durchzuführen, um die Entscheidung zwischen VV und VA ECMO entsprechend stellen zu können. Es ist festzuhalten, dass bei pulmonalem Versagen und ggf. auch höher dosierten Katecholamingaben, ansonsten aber mehr oder weniger erhaltener Links- und Rechtsventrikelfunktion *nicht* notwendigerweise eine VA ECMO indiziert ist.

### Entscheidungen für die Kanülierungsstrategie

Die Kanülierungsstrategie ist wie bei Nicht-COVID-19-Patienten an das klinische Szenario anzupassen. Bei Patienten im kardiogenen Schock oder unter Reanimation ist grundsätzlich ein perkutanes Implantieren im Bereich der Leistengefäße (V. und A. femoralis) zu empfehlen.

Eine Beinperfusionskanüle ist in allen Fällen dringend anzuraten. Aufgrund der Tatsache, dass die meisten COVID-19-Patienten eine respiratorische Beteiligung aufweisen, ist davon auszugehen, dass ein beträchtlicher Anteil der Patienten aufgrund des Harlekin-Effektes eine ungenügende Oxygenierung der oberen Körperhälfte aufweist [13]. Daher ist im Falle einer femorofemoralen VA ECMO die Oxygenierung der oberen Körperhälfte engmaschig zu überprüfen (Sättigungsmessung rechte obere Extremität). Im Falle einer schlechten Oxygenierung ist frühzeitig eine Änderung des ECMO Set-ups zu empfehlen. Gerade bei COVID-19-Patienten ist, um ein potenzielles Infektionsrisiko so gering wie möglich zu halten, ein „upgrading“ auf eine V-VA ECMO die erste Variante. In diesem Fall wird über die Jugularvene eine weitere Kanüle eingebracht, die an den arteriellen (rückführenden) Schenkel konnektiert wird. Alternative ist die Umkanülierung von der arteriellen Rückführung von femoral auf die A. subclavia. Da hierfür auch chirurgische Kapazitäten benötigt werden, ist diese Entscheidung auch an das Vorhandensein herzchirurgischer Operationskapazitäten in der Pandemiezeit zu knüpfen.

Bezüglich der unterstützenden Bildgebung bei der Implantation ist anzumerken, dass prinzipiell die transösophageale Echokardiographie die etablierteste und am häufigsten verwendete Methode ist. Da das TEE eine aerosolbildende Maßnahme darstellt, ist diese Entscheidung jedoch situationsabhängig zu fällen und gegen ein potenzielles Infektionsrisiko abzuwägen. Alternativ kann auf eine transthorakale bzw. abdominelle Ultraschallbildgebung zurückgegriffen werden.

### Kreislaufversagen an der VV ECMO

Es ist auch zu erwarten, dass ein Anteil der Patienten mit primär respiratorischem Versagen und VV ECMO im Verlauf ein Rechtsherzversagen (ARDS, hoher PEEP) oder Linksherzversagen (Myokarditis) entwickeln wird. Aus diesem Grund empfehlen wir regelmäßiges, zumindest einmal tägliches, echokardiographisches Monitoring. Für diesen Fall sind zwei mögliche Vorgehensweisen anzudenken. Welche davon für den individuellen Fall vorteilhaft ist, sollte vom jeweiligen Dienstteam entschieden werden:

- *Variante 1:* Wechsel auf V-VA mit einem zusätzlichen arteriellen Schenkel aus dem rückführenden Teil der ECMO in eine A. femoralis (s. oben).
- *Variante 2:* Umkanülierung von VV auf VA durch Kanülierung der A. subclavia (s. oben).

Die Entscheidung, welche Variante bevorzugt wird, ist aufgrund der zur Verfügung stehenden Gefäßzugänge wie auch des Patientenzustandes (extubiert vs. intubiert) zu treffen und auch auf die Erfahrung des jeweiligen im Dienst befindlichen Teams abzustimmen.

### Sonderfall: extrakorporale kardiopulmonale Reanimation (eCPR)

Im AKH Wien besteht eines der größten und auch erfolgreichsten eCPR-Programme Europas. Dennoch sind gerade in diesem Bereich gute Indikationsstellungen essenziell, um mit den vorhandenen Ressourcen gut umzugehen und um die Patienten zu identifizieren, die von dieser Maßnahme profitieren können. Entsprechend dieser Vorgabe hat die Universitätsklinik für Notfallmedizin bereits die in Tab. 2 aufgelisteten Kriterien für eine potenzielle eCPR bei Nicht-COVID-19-Patienten erarbeitet [14].

**Table 2:** 

Ziel: Eintritt Herzstillstand bis Übergabe im Krankenhaus unter 60 min wenn:
Beobachteter Herzkreislaufstillstand
Basic Life Support oder Eintreffen Rettungsdienst <5 min
Alter <70 Jahre
Schockbarer Erstrhythmus oder anyROSC-Phasen
Body-Mass-Index <35 (Leisten sind beidseits frei zugänglich)
End-Tidal-Kohlendioxid (etCO_2_) anhaltend >14 mm Hg
Pupillen nicht weit/entrundet
Keine bekannte terminale Grunderkrankung
Keine bekannte schwere peripheren arteriellen Verschlusskrankheit

Das Risiko einer Infektion des medizinischen Personals im eCPR-Setting ist als hoch einzuschätzen („aerosol generating procedure“ [AEP]). Dies gilt für nachgewiesen SARS-CoV-2-positive Patienten wie auch für alle Verdachtsfälle.

Bei eCPR SARS-CoV-2-positiver Patienten ist dringend die frühzeitige (prähospitale) Kontaktaufnahme mit dem Team der Notfallaufnahme einzufordern, damit eine Entscheidung über die ECMO-Implantation noch vor Eintreffen der Patienten im Krankenhaus getroffen werden kann. Es muss vermieden werden, SARS-CoV-2-positive Patienten für eCPR ins Zentrum zu bringen, um dann erst vor Ort festzustellen, dass sich diese nicht dafür qualifizieren. In diesem Fall würde das Infektionsrisiko für das Personal in Kauf genommen ohne Aussicht, den Patienten erfolgreich behandeln zu können. Bezüglich der Schutzmaßnahmen ist derzeit bei jeglichen unter laufender Reanimation kommenden Patienten mit unbekanntem SARS-CoV-2-Status so vorzugehen, als ob der Patient positiv wäre.

## Entscheidung über Therapiebeendigung bzw. weiterführende Therapie

Wie bei allen ECMO-Patienten ist eine Evaluierung des Patientenzustandes auf täglicher Basis notwendig. Auch hier ist auf die protektiven Maßnahmen für das medizinische Personal hinzuweisen. Im Falle von pulmonaler und/oder kardialer Recovery ist ein entsprechendes Weaning-Protokoll einzuleiten.

Sollte es zu keiner Erholung kommen, muss auch bei COVID-19-Patienten eingeschätzt werden, ob diese sich für weiterführende Therapien qualifizieren (VAD oder Transplantation). Aus derzeitiger Sicht ist zwar nicht davon auszugehen, dass dies auf einen großen Teil der Patienten zutrifft, allerdings sollte diese Option gerade bei jungen Patienten nicht von vornherein ausgeschlossen werden.

Aufgrund von wissenschaftlichen Vorarbeiten wissen wir, dass bereits ab dem 7. Tag an der VA ECMO das Überleben der Patienten deutlich abnimmt. Dies bedeutet zwar nicht, dass ab dem 7. Tag jegliche ECMO-Therapie beendet werden sollte. Allerdings ist es nötig, Therapieziele nach einer Woche Behandlungsdauer zu reevaluieren und Entscheidungen entsprechend anzupassen [15, 16].

Im Falle der nicht vorhandenen Optionen für eine weiterführende Therapie müssen eine Weiterführung der ECMO-Therapie und der Therapieabbruch diskutiert werden. Bei respiratorischer Indikation (VV ECMO) gibt es eine Subgruppe von Patienten mit Recovery auch nach deutlich längerer Zeit, sodass im Einzelfall (unter Berücksichtigung des Verlaufes und zusätzlicher Organversagen) entschieden werden muss, ob die Fortführung der ECMO-Therapie als zielführend erachtet werden kann [17, 18]. Auch diese Entscheidungen werden abhängig von der Ressourcenverfügbarkeit dynamisch beeinflusst werden.

## References

[1] Grasselli G, Pesenti A, Cecconi M (2020). Critical care utilization for the COVID-19 outbreak in Lombardy, Italy: early experience and forecast during an emergency response. JAMA.

[2] Wang D, Hu B, Hu C, Zhu F, Liu X, Zhang J, Wang B, Xiang H, Cheng Z, Xiong Y (2020). Clinical characteristics of 138 hospitalized patients with 2019 novel Coronavirus-infected pneumonia in Wuhan, China. JAMA.

[3] Huang C, Wang Y, Li X, Ren L, Zhao J, Hu Y, Zhang L, Fan G, Xu J, Gu X (2020). Clinical features of patients infected with 2019 novel coronavirus in Wuhan, China. Lancet.

[4] Hu H, Ma F, Wei X, Fang Y (2020). Coronavirus fulminant myocarditis saved with glucocorticoid and human immunoglobulin. Eur Heart J.

[5] ELSO (2020) ELSO guidance document: ECMO for COVID-19 patients with severe cardiopulmonary failure. https://www.elso.org/. Zugegriffen: 24.03.2020 10.1097/MAT.0000000000001173 PMC7273858 32243267

[6] Ramanathan K, Antognini D, Combes A, Paden M, Zakhary B, Ogino M, MacLaren G, Brodie D, Shekar K (2020). Planning and provision of ECMO services for severe ARDS during the COVID-19 pandemic and other outbreaks of emerging infectious diseases. Lancet Respir Med.

[7] Wiener Krankenanstaltenverbund (2020) Schutzbekleidung bei Corona Virus SARS-COV-2/COVID-19. https://info.wienkav.at/. Zugegriffen: März 2020

[8] Combes A, Hajage D, Capellier G, Demoule A, Lavoue S, Guervilly C, Da Silva D, Zafrani L, Tirot P, Veber B (2018). Extracorporeal membrane oxygenation for severe acute respiratory distress syndrome. N Engl J Med.

[9] Papazian L, Aubron C, Brochard L, Chiche JD, Combes A, Dreyfuss D, Forel JM, Guerin C, Jaber S, Mekontso-Dessap A (2019). Formal guidelines: management of acute respiratory distress syndrome. Ann Intensive Care.

[10] Fan E, Del Sorbo L, Goligher EC, Hodgson CL, Munshi L, Walkey AJ, Adhikari NKJ, Amato MBP, Branson R, Brower RG (2017). An Official American Thoracic Society/European Society of Intensive Care Medicine/Society of Critical Care Medicine clinical practice guideline: mechanical ventilation in adult patients with acute respiratory distress syndrome. Am J Respir Crit Care Med.

[11] Serpa Neto A, Schmidt M, Azevedo LC, Bein T, Brochard L, Beutel G, Combes A, Costa EL, Hodgson C, Lindskov C (2016). Associations between ventilator settings during extracorporeal membrane oxygenation for refractory hypoxemia and outcome in patients with acute respiratory distress syndrome: a pooled individual patient data analysis: mechanical ventilation during ECMO. Intensive Care Med.

[12] Schmidt M, Pellegrino V, Combes A, Scheinkestel C, Cooper DJ, Hodgson C (2014). Mechanical ventilation during extracorporeal membrane oxygenation. Crit Care.

[13] Lotz C, Ritter O, Muellenbach RM (2014). Assisted beating of the ischemic heart: how to manage the pulseless ST—segment-elevation myocardial infarction patient. Circulation.

[14] Poppe M, Schriefl C, Steinacher A, Clodi C, Warenits AM, Nürnberger A, Hubner P, Holzer M, Horvat J, Wiedemann D (2020). Extracorporeal cardiopulmonary resuscitation at the emergency department: a retrospective patient selection evaluation. Eur J Anaesthesiol.

[15] ELSO (2017) ELSO general guidelines for all ECLS cases. https://www.elso.org. Zugegriffen: August 2017

[16] Pichler P, Antretter H, Dunser M, Eschertzhuber S, Gottardi R, Heinz G, Pölzl G, Pretsch I, Rajek A, Wasler A (2015). Use of ECMO in adult patients with cardiogenic shock: a position paper of the Austrian Society of Cardiology. Wien Klin Wochenschr.

[17] Posluszny J, Rycus PT, Bartlett RH, Engoren M, Haft JW, Lynch WR, Park PK, Raghavendran K, Napolitano LM (2016). Outcome of adult respiratory failure patients receiving prolonged (≥14 days) ECMO. Ann Surg.

[18] Camboni D, Philipp A, Lubnow M, Bein T, Haneya A, Diez C, Schmid C, Müller T (2011). Support time-dependent outcome analysis for veno-venous extracorporeal membrane oxygenation. Eur J Cardiothorac Surg.

[19] De Geer L, Fredrikson M, Tibblin AO (2020). Frailty predicts 30-day mortality in intensive care patients: a prospective prediction study. Eur J Anaesthesiol.

